# Correction: Calcium specificity signaling mechanisms in abscisic acid signal transduction in *Arabidopsis* guard cells

**DOI:** 10.7554/eLife.10328

**Published:** 2015-07-29

**Authors:** Benjamin Brandt, Shintaro Munemasa, Cun Wang, Desiree Nguyen, Taiming Yong, Paul G Yang, Elly Poretsky, Thomas F Belknap, Rainer Waadt, Fernando Alemán, Julian I Schroeder

Brandt B, Munemasa S, Wang C, Nguyen D, Yong T, Yang PG, Poretsky E, Belknap TF, Waadt R, Alemán F, Schroeder JI. 2015. Calcium specificity signaling mechanisms in abscisic acid signal transduction in *Arabidopsis* guard cells. *eLife* **4**:e03599. doi: 10.7554/eLife.03599

Published 20 July 2015

In the originally published version of Figure 3—figure supplement 3A, the top right panel was blank. This was an error that occurred in the file format conversion, as this panel should have shown an additional in-gel kinase auto-radiography, as correctly described in the paper.

The article has been corrected.

