# Supplementary material for: Calcium specificity signaling mechanisms in abscisic acid signal transduction in Arabidopsis guard cells
Source: eLife. 2015 Jul 20;4:e03599. doi: 10.7554/eLife.03599 (PMC4507714; doi:10.7554/eLife.03599)
Supplement: Figure 6—source data 1. — The Student's t-test was used to calculate all p-values. DOI: http://dx.doi.org/10.7554/eLife.03599.018 [file elife03599s001.docx]

**Brandt et al.; Figure 6–source data 1**

Table1:

| Genotype | n of Control | n of ABA | p-value for control vs. ABA |
| --- | --- | --- | --- |
| Col0 | 6 | 6 | P<0.037 (*) |
| *slac1-1* | 3 | 3 | P=0.843 (ns) |
| *slac1-1* SLAC1-WT-1 | 4 | 6 | P<0.041 (*) |
| *slac1-1* SLAC1-WT-2 | 5 | 5 | P<0.047 (*) |
| *slac1-1* SLAC1-S59A-1 | 5 | 6 | P<0.018 (*) |
| *slac1-1* SLAC1-S59A-2 | 3 | 4 | P<0.008 (*) |
| *slac1-1* SLAC1-S120A-1 | 5 | 5 | P<0.040 (*) |
| *slac1-1* SLAC1-S120A-2 | 5 | 6 | P<0.046 (*) |
| *slac1-1* SLAC1-S59A/S120A-1 | 3 | 5 | P=0.714 (ns) |
| *slac1-1* SLAC1-S59A/S120A-2 | 7 | 10 | P=0.268 (ns) |

Table 2:

| Genotype | p-value for control vs. ABA |
| --- | --- |
| Col0 | P<0.005 (*) |
| *slac1-1* | P=0.285 |
| *slac1-1* SLAC1-WT-1 | P<0.039 (*) |
| *slac1-1* SLAC1-WT-2 | P<0.016 (*) |
| *slac1-1* SLAC1-S59A-1 | P<0.025 (*) |
| *slac1-1* SLAC1-S59A-2 | P<0.001 (*) |
| *slac1-1* SLAC1-S120A-1 | P<0.018 (*) |
| *slac1-1* SLAC1-S120A-2 | P<0.032 (*) |
| *slac1-1* SLAC1-S59A/S120A-1 | P=0.917 (ns) |
| *slac1-1* SLAC1-S59A/S120A-2 | P=0.518 (ns) |

**Figure 6-source data 1:** Statistical data and number of repeats (n) for the (**Table 1**) patch clamp measurements shown in Figure 6G and Figure 6-figure supplement 3A and (**Table 2**) for measurements of stomatal apertures presented in Figure 6H and Figure 6-figure supplement 3B (n=3 experiments and >45 total stomata per group). The student’s t-test was used to calculate all p-values.
